# Atlanta Academy of Medicine

**Published:** 1873-02

**Authors:** Jas. B. Baird

**Affiliations:** Reporter


### ATLANTA ACADEMY OF MEDICINE.

JAS. B. BAIRD, M.D., REPORTER.

[*Extract from Minutes, December* 9, 1872—*Dr. W. S. Armstrong in the Chair.*]

Dr. A. K. Smith, of the United States army, reported a case of cyanosis, as follows:

On Friday last was called to see a soldier's wife in labor. She calculated that she had passed the two hundred and eightieth day. The size of the abdomen indicated that she had arrived at full term. The labor was tedious during the forenoon, but toward evening it hurried up; a copious discharge of waters took place, immediately followed by a footling child and the placenta. The uterus contracted firmly. The child's respiration was difficult, and its color livid. It was put in cold water, ammonia applied to its nostrils, and then laid on its right side; relief immediately followed, and the mother was instructed to keep it in that position. The next morning, its position having been changed, found it livid and struggling for breath. Again placed it on the right side; the relief, as before, was immediate—the breathing becoming easy, and the natural red color taking the place of the purple. It is doing well to-day. Weighs only five pounds. The mother has had four children—all small, and were either born dead or died soon after birth.

Dr. A. W. Griggs.—Has seen a number of similar cases. The children are generally delicate, and ordinarily of premature birth. He has usually succeeded in affording relief by adopting the measures proposed by Dr. Smith. Has in mind a healthy boy, rather small. Soon after delivery, while being washed, the skin became purple, and he commenced gasping. He was placed on his right side, at an angle of fifteen degrees. Relief soon followed.

Another case—the child of a lady whose children always had this trouble; her first child premature—seven months. It was relieved by the position above described. It was avoided in the second by adopting the position as a prophylactic measure. The third was treated in the same way, but on the second day the nurse forgot the side and placed it on the left. It was soon livid and gasping. A change in the position at once corrected the unpleasant symptoms. Thinks the foramen ovale does not close up immediately after birth.

Dr. Love's experience coincides with Dr. Griggs'. Has repeatedly seen the greatest benefit follow the position on the right side. Always cautions mothers, with delicate children, on this point. His invariable rule is to place the child on the right side. Not only is the gravitation of the valve thus obtained, but also the pressure of the supernatant blood.

Dr. W. F. Westmoreland.—Has never met with a case in his practice. But once saw a case with another physician in which it was necessary to keep the patient on his right side to render him at all comfortable.

Dr. W. S. Armstrong.—Has seen two cases. The first was relieved immediately upon being placed on the right side, but finally died. The second—a seven months' child, very feeble. Respiration established with difficulty by placing it alternately in warm and cold water. It soon became cyanotic, which was relieved, as in the other instance, by a position on the right side.

Dr. Griggs called attention to the fact that nurses frequently apply the band, for retaining the dressings to the umbilicus, so tight as to obstruct respiration. He is in the habit of using adhesive plaster for this purpose.

Dr. W. F. Westmoreland presented for inspection a fibroplastic tumor removed from the thigh of a gentleman about forty years of age. It had given him no pain and but little inconvenience until recently. It was situated above the external condyle, between the vastus externus and the biceps muscles. The fascia lata was apparently inserted into the tumor. Its upper portion was almost of cartilaginous hardness—squeaking under the knife.

He also reported a rare case of tumor of the inferior maxillary bone, on right side, in a negro girl aet. eight years, about the size and shape of a hen's egg. No pain. Had existed for about a year. Some pathologists apply the term fibro-cystic to tumors in this situation; others designate them as dentigenous cyst. He thinks they both exist. This is one of the latter variety. In these tumors every portion of the bone is sometimes as thin as paper. This specimen is more recent, and consequently thicker, though so thin that in removing, it was cut away by a cartilage knife, no saw being required. The teeth were perfect. After removal found the cyst filled with serum and partially organized fibrin, in which was imbedded a tooth lying parallel with the bone.

*Operation.*—After dissecting the soft parts from the tumor, the outer bony wall was removed as before described, and the cavity packed with lint, and left to granulate. These cysts are sometimes met with, filled with serum and fibrin, and containing no tooth.

Dr. Love reported several cases of influenza which seemed to have a tendency to affect bronchial tubes and lungs.

Dr. W. F. Westmoreland.—Has seen a large number of similar cases. Has observed for nine years past that whenever we have an influenza we are apt to have an epidemic of meningitis, and he now predicts that that disease will prevail during the winter.

---

*Extract from Minutes, February* 10, 1873—*Dr. J. P. Logan, President, in the Chair.*

Dr. A. W. Griggs reported a case of convulsions in a child aged two years, from indigestion. When first seen she was unconscious; eyes fixed forward; muscles rigid; soon convulsive movements of one side occurred, and after a few minutes, convulsive movements of the other side. Thus there was no relaxation; respiration was difficult; feared danger from asphyxia; head was slightly drawn down on one side or the other. During the convulsive movements of the right side, the eyes were turned obliquely toward the left, and when convulsions occurred on the opposite side, they looked obliquely toward the right. Regarded the disturbance as reflex action, due to intestinal irritation.

As soon as the child could swallow, administered a powder of calomel and ipecac. It produced no emesis. The action of the heart was inordinate; pulse very rapid. Injected hypodermically one drop of veratrum viride, which diminished cardiac action. She also had a mustard bath, and sinapisms to the spine, neck and extremities. Attempted to administer chloroform by inhalation, but was not successful; owing to the impeded respiration, the attempt was not persisted in. Ten drops of chloroform in camphor water was then introduced per rectum. Relaxation followed, and consciousness speedily returned. An enema of castor oil and warm water produced an evacuation from the bowels, containing a large quantity of pop-corn, the cortex of which remained intact. The child had also eaten imprudently of candy and nuts. A second enema was given with the same results, and recovery was complete in a few hours.

Dr. W. F. Westmoreland reported a fatal case of meningitis in a lady aged forty-five. The attack was insidious in its approach. The symptoms were not alarming. She had recently left a community where the disease was prevailing to an alarming extent. She died suddenly, on the fourth day, her symptoms to within a few hours of death being considered favorable—there being apparently a marked improvement in her condition in every respect. She was treated by a blister to the spine, a cathartic, opium and quinine. The cathartic he thought acted injuriously; the opium seemed to benefit decidedly.

A long discussion ensued, in which Dr. W. F. Westmoreland stated that it was his belief that the so-called cerebro-spinal meningitis was an inflammation of the meninges of the brain and spinal cord, differing from ordinary idiopathic meningitis, and due to a peculiar, specific and atmospheric poison, which sometimes proves fatal by the shock inflicted upon the system, and in these cases no post-mortem anatomical lesions are presented. He holds, therefore, that the treatment adopted for traumatic and ordinary inflammations is not appropriate to the disease under consideration.

[*Extract from Minutes, February* 17, 1873—*Dr. J. P. Logan, President, in the Chair.*]

Dr. Connally reported the case of a young man who resides forty miles from the city. Had spent the day here attending to business. Early in the evening he entered a car to return home, when he was attacked with violent pain in the head and a feeling of general distress. He immediately returned to the hotel, and the doctor was summoned.

Found him retching and vomiting; seemed incoherent, and expressed great anxiety and alarm. Extremities cool; pulse normal in rate, though small and wiry; integument presented a congested appearance; pupils dilated.

Prescribed one-fourth of a grain of morphia, which was repeated in fifteen or twenty minutes. A sinapism with capsicum was applied to the spine, and forty grains of calomel were administered. A consultation was held, and bleeding decided on. He refused to submit to venesection. Two hours and a half had elapsed since the attack commenced, and reaction had begun to take place. The pulse remained the same, and he expressed himself somewhat relieved, though he continued in a semi-delirous condition, and pupils remained dilated. Wet cups were then applied to the back of his neck, and twelve or fourteen ounces of blood abstracted. The pulse immediately improved; the calomel acted satisfactorily the next morning, and he returned home at eight o'clock in the evening, feeling well. No evidence of excessive indulgence in the use of alcoholic liquors was furnished. Supposed to be a case of cerebro-spinal meningitis.

Dr. W. F. Westmoreland reported two cases of "so-called cerebro-spinal meningitis." The first, a lady aged thirty, had suffered with influenza. Called at two o'clock Wednesday night last. Circulation so rapid, pulse could scarcely be felt; terrific pain in the head; vomiting freely; more or less muscular rigidity; slightly incoherent; skin presented a tallowy hue, due to congestion of the cutaneous capillaries. Opium and quinine were given, and improvement began in a few hours. Dismissed the case well next day. The day following at 6 o'clock P.M., was called to see a lad aged ten. Presented exactly the same symptoms as did the former case. The same treatment was adopted. At nine o'clock the next morning, seemed decidedly better, but died suddenly at one o'clock in the afternoon.

Dr. John G. Westmoreland reported the following case:

A man aged about forty years, robust, about Christmas was exposed to cold, and after riding four miles in the night, experienced an uncomfortable sensation of tightness across his chest, and difficulty of breathing. It continued for an hour or two, and gradually passed off, but recurred from time to time. Finally it became so troublesome that it was necessary to give up his business, and only obtained relief by leaning forward resting his head upon a table; being unable to lie down for fear of suffocation.

This was his condition when seen about ten days ago. The heart was pulsating violently, though regularly—about ninety or one hundred beats per minute. Symptoms of engorgement of the liver, or perhaps sub-acute hepatitis, were present, as evinced by tenderness and weight of that organ, and dullness on percussion below the ribs. This was relieved by a blister over the liver, but as the other symptoms continued, he looked upon the heart as the sole cause of the disturbance.

Ordered ten drops of digitalis three times a day, and wet cups over the heart; opium as required by restlessness. Dyspnœa relieved, but the inordinate action of the heart continued, though irregularly and intermittently. One week ago yesterday, and two weeks after the beginning of the attack, he failed to pronounce words, and soon after, while sitting up, he suddenly lost the use of his left arm and leg. The next day he recovered the use of his limbs. Has been irrational a portion of the time. The slightest movement causes the heart to intermit every other beat. Had a dry cough in the early stages of the attack. Has not been an intemperate man. The action of the heart is now irregular—flutters from slightest movement; mind not so clear; restless and anxious. The points of interest are so much disturbance of the heart and no pain, and the cause of the paralysis. He has never suffered from rheumatism, and at no time was any uneasiness discernible upon consulting the heart.

*Extract from Minutes, February* 24, 1873—*Dr. J. P. Logan, President, in the Chair.*]

The President, Dr. Logan, desired to call attention to the treatment of a troublesome feature of sub-involution of the uterus. He refers to menorrhagia or metrorrhagia, which, in his experience, has been one of the most troublesome features that he has ever had to contend with. The plan of treatment is probably not original, but in his hands has proved so satisfactory that he thinks he is warranted in reporting it. It consists simply in the application of the official tincture of iodine to the inner surface of the uterus by means of the cloth tent introduced to the profession by Dr. Taliaferro, of this city. The tent is saturated with the tincture, then dipped in glycerine and introduced fully into the cavity of the uterus, where it is allowed to remain for twenty-four hours. This process is repeated every third, fifth or seventh day according to circumstances. He believes that the treatment is also well adapted to other forms of *hemorrhage* from the uterus. In this mode of treatment he has great confidence. He has, in a few cases, found improvement follow the topical use of muriated tincture of iron, but it is by no means so satisfactory as the treatment just referred to. Knows of no contribution to gynæcology, during the last year, of more importance than that of the cloth tent for the introduction of remedial agents into the cavity of the uterus.

Dr. Taliaferro remarked that he thought this treatment of hemorrhage from sub-involution was original with the Doctor. It was a peculiar hemorrhage, due to the perversion of a physiological process, and not to a granular or inflammatory condition of the lining membrane or to metritis. He has treated sub-involution with iodine and had the hemorrhage to cease, but it never occurred to him that it was due to the iodine.

Dr. W. F. Westmoreland stated that he desired to report a case, such as had never before been reported to the Academy, and he hoped never would be again—a death from chloroform. He, in company with Drs. D'Alvigny, Johnson and Simpson, this afternoon, repaired to the rooms of Mr. ——, a gentleman aged thirty-five, a farmer, robust and apparently in fine health, for the purpose of performing an operation upon the eye. At the request of the patient, the administration of chloroform was commenced, and in a few minutes death resulted. As Dr. J. T. Johnson had charge of the anesthetic, he requested him to continue the report.

Dr. Johnson responded by saying that the patient was placed in a comfortable recumbent position and his clothing carefully loosened. The chloroform was administered in the usual way, by means of a towel folded in the form of a cone. Care was observed to allow an abundant admixture of atmospheric air. After a few inspirations the breathing became stertorous, when the chloroform was withheld, the Doctor remarking that he did not like his breathing; this, however, passed off at once, and the towel was re-applied, the respiration and pulse being closely watched. The operation was then commenced, but the patient not being fully under the influence of the anesthetic, offered resistance and complained of pain. In a few moments the respiration again became stertorous, the face livid, and an occasional violent spasmodical closure of the mouth. The chloroform was immediately withdrawn, and Dr. Westmoreland's attention called to the circumstance. He, remarking that the patient had "swallowed his tongue," introduced a tenaculum and drew the tongue forward, saying he is all right now but the operation must be completed without chloroform. The breathing immediately improved, but continued only a few seconds, when, after a few gasps, it ceased entirely. Energetic measures for his resuscitation were at once adopted. The doors and windows were thrown open, electricity was used, and, at the same time, the usual methods of artificial respiration were vigorously employed and continued, with friction to the lower extremities, for two and a half or three hours, but without avail. The time, from the commencement of the inhalation to the final withdrawal of the chloroform, did not exceed five minutes. The amount employed was between two and three drachms.

Dr. Westmoreland added that the heart continued to beat faintly for twenty minutes after the respiration ceased; furthermore, that he had used the same chloroform a few days before in a case of amputation of the leg, and the patient came out from it badly. He had left a specimen of it with a chemist for analysis. This is the first patient he had ever lost from chloroform.

